# Is low back pain related to the body composition, flexibility, and postural deviations in rural workers?

**DOI:** 10.47626/1679-4435-2022-983

**Published:** 2024-08-05

**Authors:** Marcelo Henrique Glänzel, Guilherme Görgen da Rocha, Analie Nunes Couto, Valeriano Antônio Corbelini, Miriam Beatrís Reckziegel, Hildegard Hedwig Pohl

**Affiliations:** 1 Departamento de Ciências da Saúde, Universidade de Santa Cruz do Sul, Santa Cruz do Sul, RS, Brazil; 2 Escola de Educação Física, Fisioterapia e Dança, Universidade Federal do Rio Grande do Sul, Porto Alegre, RS, Brazil; 3 Centro de Educação Física e Desportos, Universidade Federal de Santa Maria, Santa Maria, RS, Brazil; 4 Escola de Medicina, Pontifícia Universidade Católica do Rio Grande do Sul, Porto Alegre, RS, Brazil

**Keywords:** rural health, occupational health, back pain, body composition, posture, saúde rural, saúde ocupacional, dor lombar, composição corporal, postura

## Abstract

**Introduction:**

Hard work in the countryside can lead to the onset of pain conditions, which in turn trigger different degrees of labor reduction and musculoskeletal disorders. Low back pain is one of the most common disorders that lead to inactivity, and obesity seems to be associated with the development of low back pain symptoms, since abdominal fat causes mechanical demands in this region due to excessive load.

**Objectives:**

To analyze low back pain and its relationship with body composition, flexibility, and posture in rural workers.

**Methods:**

Rural workers (n = 55) were grouped according to the presence of low back pain or absence of low back pain symptoms. Body composition, flexibility, and posture were assessed and compared between groups. A principal component analysis was used to group variables to identify possible associations among variables and low back pain.

**Results:**

The low back pain group presented greater obesity rates than the group without symptoms. Regarding low back pain prevalence, most of the participants had pain symptoms and showed postural deviations. Principal components analysis showed that the group without symptoms was mainly related to the amount of muscle tissue, while the low back pain group was to the adipose tissue.

**Conclusions:**

Low back pain appears to be associated with body composition and postural deviations, while musculoskeletal and adipose tissues are protective and risk factors for low back pain, respectively, in rural workers.

## Abstract (pt)

**Introdução:**

O trabalho árduo no campo pode levar ao aparecimento de quadros de dor, que desencadeiam diferentes graus de redução do trabalho e distúrbios musculoesqueléticos. A dor lombar é um dos principais distúrbios que levam a inatividade. A obesidade parece estar associada a dor lombar, pois a gordura abdominal provoca demandas mecânicas nessa região devido à carga excessiva.

**Objetivos:**

Analisar a dor lombar e sua relação com a composição corporal, flexibilidade e postura em trabalhadores rurais.

**Métodos:**

Trabalhadores rurais (n = 55) foram agrupados de acordo com a presença ou ausência de sintomas. A composição corporal, flexibilidade e postura foram avaliadas. Uma análise de componentes principais foi utilizada para identificar possíveis associações entre as variáveis e a dor lombar.

**Resultados:**

O grupo com dor lombar apresentou maiores taxas de obesidade do que o grupo sem sintomas. Em relação à prevalência de dor lombar, a maioria dos participantes apresentava dor e desvios posturais. A análise de componentes principais mostrou que o grupo sem sintomas estava relacionado à quantidade de tecido muscular, enquanto o grupo com dor lombar estava ao tecido adiposo.

**Conclusões:**

A dor lombar parece estar associada à composição corporal e desvios posturais, enquanto os tecidos musculoesquelético e adiposo são fatores protetores e de risco para dor lombar, respectivamente, em trabalhadores rurais.

## INTRODUCTION

In Brazil, approximately 18% of the workers work in the countryside.^1^ Rural workers’ health status is directly or indirectly influenced by factors that include working conditions, lifestyle, diet, and social relationships.^2^ Also, behavioral factors of the rural population are correlated with lower income and education levels.^3^ In agriculture, care for the work environment and its optimization is little observed due to the fragmented nature of this activity, the reduced possibilities of this population’s collective organization, and it is also related to the large territory in which the production units are located.^4^

Hard work in the countryside can lead to the onset of pain conditions, which in turn trigger different degrees of labor reduction and musculoskeletal disorders.^5^ Low back pain (LBP) (i.e., acute or chronic pain in the lumbar or sacral regions) is one of the most common musculoskeletal disorders that lead to inactivity, postural disorders, and muscle dysfunctions, which can result in disability, reduced quality of life, and loss of productivity at work.^6^ In rural workers, LBP is the most commonly reported complaint, having significant consequences on both the clinical and economic statuses of these individuals.^7^

There is an increasing prevalence of LBP in Brazil, which has shown a 79% increase in the total number of years lived with disability since 1990.^8^ In rural workers, the annual prevalence of LBP reaches 74% in Nigeria,^9^ 58% in Canada,^7^ and 56% in Thai^10^; however, in Brazilian workers evidence is scarce. When compared to workers from other economic sectors, rural workers demonstrate greater exposure to LBP risk factors,^7^ and have a longer time off work due to LBP,^8^ since work in the countryside consists of strenuous tasks and many manual demands,^11^ such as exposure to vibrations, trunk flexion and rotational movements performed repeatedly, and lifting/carrying high loads at heights above the shoulder joint.^6,10^

The presence of LBP contributes to physical inactivity and decreased muscle mass (MM) and strength.^12^ The lack of physical activities contributes to the development of obesity, which is considered a public health trouble.^12,13^ Previous evidence^12,14^ suggested that individuals with increased body fat levels tended to have an increased risk of developing LBP. Thus, obesity seems to be associated with the development of LBP,^13^ since abdominal fat causes mechanical demands in this region due to excessive load, generating structural changes and painful conditions.^15^ Due to its multifactorial nature, obesity may be related to chronic diseases, postural inadequacies related to the work environment, inactivity, and biomechanical issues.^16^ Thus, knowing the role and relationship of these factors can help to develop strategies to reduce the emergence of LBP and time off work in rural workers.

Considering the aspects addressed and the limited number of available studies in rural workers’ health, and understanding the importance of musculoskeletal disorders in this population,^11^ this study aimed to analyze the presence of LBP and its relationship with body composition, flexibility, and postural deviations in rural workers.

## METHODS

### PARTICIPANTS

To participate in the study, the subjects should meet the following inclusion criteria: a) rural producers; b) age equal to or older than 18 years old; c) presenting the necessary physical conditions to perform the proposed tests. The following exclusion criteria were considered: a) presenting any pathology that could make it impossible to perform the tests. Fifty-five rural workers (25 men and 30 women) from municipalities in the southern microregion of Conselho Regional do Desenvolvimento do Vale do Rio Pardo (composed of 23 municipalities in the central-west region of the state of Rio Grande do Sul, Brazil) participated in this study. All participants met the inclusion criteria, were informed about the study, and gave written consent to participate. This study was approved by the institutional Research and Ethics Committee (Number 1.337.659; CAAE 50617815.6.0000.5343).

### STUDY DESIGN

From the lifestyle questionnaire,^17^ sociodemographic variables and the presence of LBP were investigated, with workers being dichotomized and grouped according to the presence (LBPG), or absence (NLBPG) of symptoms. To assess pain perception, the self-reported Visual Analog Scale (VAS) for pain was used, which consists of levels stratified from 0-10, with 0 corresponding to no pain and 10 corresponding to the maximum level of pain experienced by the participants, classified as: 1-3 as “mild”, 4-7 “moderate”, and 8-10 as “intense”.^18^ Soon after, assessments of body composition (anthropometry and bioimpedance analysis), lumbar region’s flexibility (sit and reach test [SRT]), and posture (New York Test [NYT]) were performed with each participant.

### BODY COMPOSITION’S ASSESSMENT

In the anthropometric assessment, the following variables were used: body mass and height, estimating the body mass index (BMI), as well as bone mass (BM), lean body mass (LBM), and MM. The body composition assessment was also performed using a bioimpedance device (In-Body 720; Biospace, Seoul, South Korea) considering the variables body fat mass (BFM), skeletal MM (SMM), percentage of body fat (%BF), and visceral fat area (VFA).

### FUNCTIONAL PARAMETERS’ ASSESSMENT

The flexibility of lumbar region was assessed from the SRT, using the Wells bench, in which the total distance reached represents the final score, with three reaching attempts performed. The highest result among the three attempts was considered for the analyses. The results of performance on the SST were stratified according to participants’ gender, and classified by the following categories: below average, average, and above average.^19^

Postural deviations were identified by photogrammetry, from the NYT.^20^ For this purpose, Nikon digital camera model D3000 was used, with a VIVITAR-series 63.7” tripod. The camera was positioned on the tripod and placed at a distance of 3 m, with a height of 1.1 m, recording the participant in the posterior and lateral views. Considered an objective method for postural assessment, six segments in the posterior plane (head, shoulders, spine, hip, feet, and plantar arch) and seven segments in the lateral plane (neck, chest, shoulders, thoracic spine, trunk, and pelvis, lumbosacral spine, and abdomen).

The scores determined to classify the deviations observed during the NYT were: scores of 5.0 points for the normal pattern; 3.0 points for moderate postural deviation; and 1.0 point for severe postural deviation in each segment. The postural classification was obtained by summing the items and considered “normal posture” as scores between 56-65 points; “moderate deviation” as those between 40-55 points; and “severe postural deviation” as those up to 39 points.^21^

### STATISTICAL ANALYSIS

Data processing and statistical analysis were performed using SPSS 23.0 (IBM Corporation, Armonk, NY, USA). Descriptive analysis was performed using frequencies and percentages, mean and standard deviation (SD). To test the data’s normality, the Shapiro-Wilk test was used. To compare the body composition parameters and the values obtained in the SST and NYT between groups, Student’s *t* test for independent samples was used for parametric variables, and the Mann-Whitney’s *U* test for non-parametric variables, considering a significance level of α ≤ 0.05. To compare the grouping of variables, principal components analysis with Varimax was used, with self-scaling per variable, Kaiser-Meyer-Olkin (KMO) normalization test per sample, in that values obtained between 0.5 and 1.0 indicate that the factor analysis is adequate, and ≤ 0.05 in Bartlett’s sphericity test so that we can perform the principal components analysis.

Models were created considering the NLBPG and LBPG (KMO = 0.554; KMO = 0.528 respectively and Barlett’s sphericity test < 0.001 for both) taking into account a factor loading ≤ 0.40 for the grouping of variables, in which each component (factor) has an explained variation of LBP and the greater the explained variation, the higher is the association between the variables and outcomes.

## RESULTS

In the evaluated rural workers, it was observed that most of these are between socioeconomic classes C1 and B2 (92.7%). Concerning the classification by length of work experience, 40% of the participants have less than 20 years of work and 60% over 20 years in this business. Regarding BMI, 29.7% of participants in the LBPG were obese versus 5.6% in the NLBPG. The VFA was high in 54.1% of LBPG and 33.3% in NLBPG. The prevalence of LBP was 67.2% in rural workers and, of those with LBP, most (91.9%) had moderate pain. Regarding flexibility, when the classifications of “average” and “above average” were analyzed together, the results are similar in both groups. In addition, 94.6% of the participants in the LBPG present postural deviations (Table 1).

**Table 1:** Demographic and general information of the included rural workers

Variables	NLBPG n (%)	LBPG n (%)	Total n (%)
Sex			
Male	8 (44.4)	17(45.9)	25 (45.5)
Female	10 (55.6)	20 (54.1)	30 (54.5)
Socioeconomic class			
C2	2 (11.1)	2 (5.4)	4 (7.3)
C1	13 (72.2)	14 (37.8)	27 (49.1)
B2	3 (16.7)	21 (56.8)	24 (43.6)
Working time (years)			
<20	8 (44.4)	14 (37.8)	22 (40.0)
>20	10 (55.6)	23(62.2)	33 (60.0)
Age group (years)			
<40	4 (22.5)	8(21.6)	12 (21.8)
40-49	5(27.8)	13 (35.1)	18 (32.7)
50-60	6 (33.3)	8(21.6)	14 (25.5)
>60	3 (16.7)	8(21.6)	11 (20.0)
BMI			
Recommended	7 (38.9)	12 (32.4)	19(34.5)
Overweight	10 (55.6)	14 (37.8)	24 (43.6)
Obesity	1 (5.6)	11 (29.7)	12 (21.8)
VFA^*^			
Normal	12 (66.7)	16 (43.2)	28 (50.9)
High	6 (33.3)	20 (54.1)	26 (47.3)
AVS			
Painless	18 (100.0)	-	18 (32.7)
Mild	-	1(2.7)	1 (1.8)
Moderate	-	34 (91.9)	34 (61.8)
Intense	-	2 (5.4)	2 (3.6)
SRT			
Above average	13 (72.2)	19 (51.3)	32 (58.2)
Average	1 (5.6)	10 (27.0)	11 (20.0)
Below average	4 (22.2)	8(21.6)	12 (21.8)
NYT			
Normal	4 (22.2)	2 (5.4)	6 (10.9)
Moderate deviations	10 (56.6)	28(75.7)	38(69.1)
Severe deviations	4 (22.2)	7 (18.9)	11 (20.0)
Total	18 (100.0)	37(100.0)	55 (100.0)

* One female missing. AVS = analogic visual scale; BMI = body mass index; LBPG = low back pain group; NLBPG = no symptoms of low back pain group; NYT = New York Test; SRT = sit and reach test; VFA = visceral fat area.

Table 2 shows the results related to the comparison in body composition, flexibility levels, and postural deviation between the LBPG and the NLBPG. No differences were found between groups in all parameters evaluated (p > 0.05).

**Table 2:** Comparison of body composition parameters, flexibility levels and postural deviations between NLBP and LBPG

Variables	NLBPG	LBPG	p-value
BM (kg)	10.35 ± 1.99	11.11 ± 1.66	0.127
LBM (kg)	54.89 ± 10.79	58.84 ± 10.59	0.857
MM (kg)	26.89 ± 6.78	28.20 ± 6.41	0.477
BFM (kg)	18.34 ± 4.45	22.20 ± 8.35	0.197
SMM (kg)	29.92 ± 7.06	31.55 ± 6.80	0.579
BFM_bia_ (kg)	19.61 ± 6.78	24.67 ± 12.04	0.233
%BF (%)	26.98 ± 8.86	30.02 ± 10.77	0.244
VFA (cm^2^)	86.91 ± 29.15	107.83 ± 44.23	0.090
SRT(cm)	28.09 ± 7.83	25.30 ± 8.89	0.351
NYT (score)	48.22 ± 7.39	45.94 ± 6.21	0.238

Data expressed as mean ± standard deviation.

* Significant difference p ≤ 0.05.

%BF = percent body fat; BFM = body fat mass; BFM_BIA_ = body fat mass by bioimpedance; BM = bone mass; LBM = lean body mass; LBPG = low back pain group; MM = muscle mass; NLBPG = no symptoms of low back pain group; NYT = New York test; SMM = skeletal muscle mass; SRT = sit and reach test; VFA = visceral fat area.

Considering the greater number of obese participants in the LBPG, principal components analysis was performed (Table 3).

**Table 3:** Analysis of principal components of body composition parameters, levels of flexibility and postural deviations of the NLBPG and the LBPG

Variables		NLBPG			LBPG	
	Factor 1		Factor 2	Factor 1	Factor 2	Factor 3
BM (kg)	0.878	0.115		-0.053	0.909	-0.153
LBM (kg)	0.981	0.033		0.188	0.975	-0.021
MM (kg)	0.928	-0.132		-0.05	0.961	0.050
BFM (kg)	-0.011	0.87		0.956	0.081	-0.115
SMM (kg)	0.950	-0.224		-0.185	0.949	-0.045
BFM_BIA_ (kg)	-0.077	0.984		0.99	0.021	-0.060
%BF (%)	-0.47	0.872		0.926	-0.337	-0.047
VFA (cm^2^)	0.044	0.982		0.949	0.042	-0.063
SST (cm)	-0.413	0.081		0.024	-0.3	0.759
NYT (score)	0.034	-0.594		-0.209	0.17	0.81
Explained variation (%)	46.02	31.87		41.52	36.35	11.86
Accumulated variation (%)	46.02	77.89		41.52	77.87	89.73

Principal component analysis; Varimax rotation method with Kaiser normalization; numbers in bold represent variables with factor loading > 0.4.

%BF = percent body fat; BFM = body fat mass; BFM_BIA_ = body fat mass by bioimpedance; BM = bone mass; LBM = lean body mass; LBPG = low back pain group; MM = muscle mass; NLBPG = no symptoms of low back pain group; NYT = New York test; SMM = skeletal muscle mass; SRT = sit and reach test; VFA = visceral fat area.

**Figure 1:**
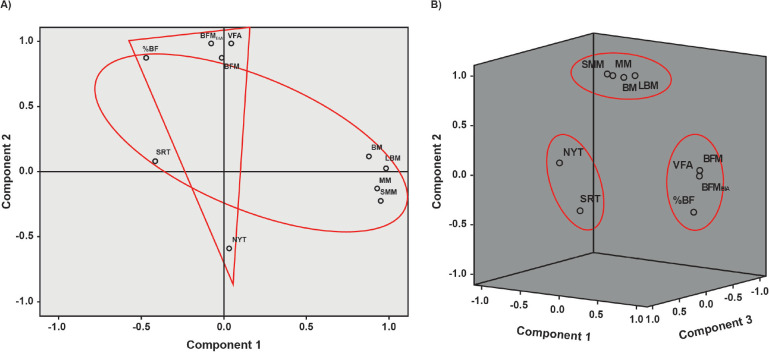
Principal component analysis diagrams with the groups’ information. %BF = percent body fat; BFM = body fat mass; BFM_BIA_ = body fat mass by bioimpedance; BM = bone mass; LBM = lean body mass; MM = muscle mass; NYT = New York test; SMM = skeletal muscle mass; SRT = sit and reach test; VFA = visceral fat area.

The principal components analysis formed different groups of variables, grouping the NLBPG into two factors representing 77.89% of the model, containing the variables BM, LBM, MM, and SMM, with a positive relationship, and %BF with a negative relationship in factor 1; the variables BFM, BFM_BIA_, and VFA were related to factor 2, in which %BF appears again with a positive association; lumbar region flexibility and postural deviations were negatively related to factors 1 and 2 (respectively). Conversely, in the LBPG, the grouping occurred in three factors representing 89.73% of the model, and the variables were grouped differently when compared to the NLBPG, with factor 1 including BFM, BFM_BIA_, %BF, and VFA; factor 2, BM, LBM, MM, and SSM; and factor 3, lumbar region flexibility and postural deviations. In the principal component analysis diagrams (Figure 1), the groups’ information can be observed.

## DISCUSSION

This study aimed to investigate the relationships between LBP symptoms and body composition, flexibility, and postural deviations in rural workers. Regarding body composition, the LBPG presented greater obesity rates and VFA values than the NLBPG. About the prevalence of LBP in rural workers, most of the participants had pain symptoms and postural deviations. Principal components analysis showed that the NLBPG was mainly related to the amount of muscle tissue, while the LBPG was to the adipose tissue.

Association between obesity and pain has already been discussed, as in the study by Deere et al.,^22^ which showed that obesity can represent an important risk factor for the occurrence and persistence of musculoskeletal pain in young adults. Stone & Broderick^23^ found a relationship between level III obesity and pain reported by individuals. In the same direction, Shiri et al.^24^ found that obese and physically inactive individuals were more likely to develop LBP. A recent systematic review^14^ suggests that excess body fat mass is the essence of the process to develop symptoms of LBP, regardless of whether the BMI is considered normal. Furthermore, with increasing body fat mass, the risk of developing LBP increases by approximately 20%.^14^

The increase in fat mass, especially the fat located in the abdominal region, would increase the gravitational load on the spine, and constant stress can induce structural changes in the intervertebral discs, resulting in local pain in the lower back.^14,15^ In addition to the biomechanical point of view, it is possible that adipose tissues, which are metabolically active, may release a large number of pro-inflammatory cytokines and substances related to metabolism, which may lead to LBP from the nerve ingrowth or neovascularization.^14^

Regarding the prevalence of LBP in rural workers, we observed that more than half of the evaluated participants had pain. These results are close to those found by Tella et al.,^9^ who obtained a rate of 74% in Nigerian workers. Other studies obtained lower values, such as those by McMillan et al.^7^ and Udom et al.^10^ who found an annual prevalence of 58% in Canada, and of 56% in Thai rural workers, respectively. Although exposure to factors related to physical work can contribute to the development of LBP, there seems to be no consensus on the body’s mechanical and physiological responses to the various types of agricultural tasks found in the routine of these workers, but is possible that high or low levels of capacity could influence the development of pain symptoms.^7^

Another highlight of our study is that most participants had pain symptoms and had postural deviations. Considering that different degrees of functional incapacity can occur due to musculoskeletal disorders, which can cause illness and worker’s withdrawal from their work activities. Since several musculoskeletal disorders can be detected, analysis of static posture is one of the steps in preparing exercisebased interventions to correct postural dysfunctions.^25^ Thus, our findings demonstrate the importance of identifying dysfunctions through postural assessment.

Our principal components analysis allowed us to observe that the grouping of variables was different between the two groups, since, for the NLBPG, the variables BM, LBM, MM, and SSM were positively related in factor 1, demonstrating that a better musculoskeletal condition may be associated with absence of LBP. This fact may be associated with the balance of body structures, maintained by the musculoskeletal system during a specific activity,^26^ and correct posture, which are important factors in preventing injuries caused by improperly performed activities.^27^ Trunk muscles, for example, play a very important role in supporting the spinal column; therefore, lower levels of MM in the trunk region may increase the risk of developing LBP, due to a possible sagittal imbalance of the spine.^28^

Our analyzes also showed a negative relationship between the %BF and the lumbar region flexibility, suggesting that decreased fat mass can be a protective factor for pain in the lumbar spine, once, as mentioned earlier, when located in the region abdominal, fat mass can cause additional structural overload in the lower back.^14,15^ About flexibility, most individuals presented above average rating levels,^19^ which could be a positive factor against LBP symptoms, since restricted flexibility of posterior chain muscles (e.g., lower back and hamstring muscles) has been linked to reduced lumbar lordosis, which in turn is associated with increased risk of developing LBP.^29^

Factor 2 grouped positively the variables referring to adipose tissue (i.e., BFM, BFM_BIA_, %BF, and VFA), and negatively the postural deviations, suggesting that posture influences the presence of pain symptoms. The two components formed for this model explain 77.89% of this association.

The variables of the LBPG were grouped differently, with the formation of three components explaining 89.73% of the model, and these were grouped as follows: in factor 1, body composition parameters referring to the adipose tissue (i.e., BFM, BFM_BIA_, %BF, and VFA); in factor 2, variables related to the musculoskeletal system (i.e., BM, LBM, MM, and SSM); and, finally, in factor 3, lumbar region flexibility and postural deviations.

In summary, the groups were similar regarding the assessed parameters; however, principal components analysis allowed us to observe that the variables related to adipose tissue were found in factor 1, presenting a possible association with the presence of LBP in the rural workers. Our findings bring new information about the rural workers’ health, reinforcing that obesity, as well as pain conditions and their characteristics, are themes that must be constantly focused, in the search for preventive measures targeted at this population.^30^ Thus, this study contributes so that professionals who deal with this symptomatology can broaden their approach, focusing on aspects related to the work process, as well as the rural workers’ health and lifestyle. New studies of high methodological quality are needed to establish new relationships, since both LBP and obesity are considered public health problems.

## CONCLUSIONS

The LBP appears to be associated with body composition and postural deviations in rural workers. A high percentage of rural workers with LBP have some level of obesity, accompanied by postural dysfunctions, while flexibility was not associated with the presence of the symptoms. In addition, the grouping of the evaluated parameters indicated that the amount of musculoskeletal tissue may be a protective factor for LBP symptoms, while excess adipose tissue seems to increase exposure to these symptoms.

## References

[1] Rocha FLR, Marziale MHP, Hong O-S. (2010). Work and health conditions of sugar cane workers in
brazil. Rev Esc Enferm USP.

[2] De Souza S, Pappen M, Krug SBF, Renner JDP, Reuter CP, Pohl HH. (2018). A narrative review associating health vulnerability and
environmental factors among rural workers. Rev Bras Med Trab.

[3] Watkins C, Macy G, Golla V, Lartey G, Basham J. (2018). The “total worker health” concept: A case study in a rural
workplace. J Occup Environ Med.

[4] Da Costa CKL, de Lucena NMG, Tomaz AF, Másculo FS. (2011). Avaliação ergonômica do trabalhador rural:
Enfoque nos riscos laborais associados à carga
física. Gest Prod Oper Sist.

[5] Truszczyńska A, Truszczyński O, Rąpała K, Gmitrzykowska E, Tranowski A. (2014). Postural stability disorders in rural patients with lumbar spinal
stenosis. Ann Agric Environ Med.

[6] Uçar İ, Karartı C, Cüce İ, Veziroğlu E, Özüdoğru A, Koçak FA (2021). The relationship between muscle size, obesity, body fat ratio,
pain and disability in individuals with and without nonspecific low back
pain. Clin Anat.

[7] McMillan M, Trask C, Dosman J, Hagel L, Pickett W. (2015). Prevalence of musculoskeletal disorders among saskatchewan
farmers. J Agromedicine.

[8] Carregaro RL, Tottoli CR, Rodrigues DDS, Bosmans JE, da Silva EN, van Tulder M. (2020). Low back pain should be considered a health and research priority
in brazil: Lost productivity and healthcare costs between 2012 to
2016. PLoS One.

[9] Tella BA, Akinbo SR, Asafa SA, Gbiri CA. (2013). Prevalence and impacts of low back pain among peasant farmers in
south-west nigeria. Int J Occup Med Environ Health.

[10] Udom C, Janwantanakul P, Kanlayanaphotporn R. (2016). The prevalence of low back pain and its associated factors in
thai rubber farmers. J Occup Health.

[11] Barneo-Alcántara M, Díaz-Pérez M, Gómez-Galán M, CarreñoOrtega Á, Callejón-Ferre Á-J. (2021). Musculoskeletal disorders in agriculture: A review from web of
science core collection. Agronomy.

[12] Kim WM, Lee SA, Park YJ, Seo YG. (2021). Effects of rehabilitation exercise on cardiovascular risk factors
and muscle cross-sectional area in overweight patients with low back
pain. Healthcare (Basel).

[13] Hershkovich O, Friedlander A, Gordon B, Arzi H, Derazne E, Tzur D (2013). Associations of body mass index and body height with low back
pain in 829,791 adolescents. Am J Epidemiol.

[14] You Q, Jiang Q, Li D, Wang T, Wang S, Cao S. (2021). Waist circumference, waist-hip ratio, body fat rate, total body
fat mass and risk of low back pain: A systematic review and
meta-analysis. Eur Spine J.

[15] Briggs MS, Givens DL, Schmitt LC, Taylor CA. (2013). Relations of c-reactive protein and obesity to the prevalence and
the odds of reporting low back pain. Arch Phys Med Rehabil.

[16] Borges CdS, Fernandes LFRM, Bertoncello D. (2013). Correlação entre alterações lombares
e modificações no arco plantar em mulheres com dor
lombar. Acta Ortop Bras.

[17] Pohl HH, Reckziegel MB, Vittiello IP, Galliano LM. (2010). Saúde do trabalhador e estilo de vida: Uma visão
multisetorial da aptidão física. FIEP Bull.

[18] Sociedade Brasileira para Estudo da Dor Hospital sem dor diretrizes para implantação da dor como
5º sinal vital.

[19] The American College of Sports Medicine (2000). Manual do ACSM para teste de esforço e prescrição
de exercício.

[20] Dos Santos JB, Moro ARP, Cezar MR, Reis PF, Luz JD, dos Reis DC. (2005). Descrição do método de
avaliação postural de portland state
university. Fisioter Bras.

[21] Conti PBM, Ribeiro MAGO, Ribeiro JD, Okuro RT, Gonçalves RM, Schivinski CIS. (2006). Alteração postural em pacientes com fibrose
cística. Rev Paul Pediatr.

[22] Deere KC, Clinch J, Holliday K, McBeth J, Crawley EM, Sayers A (2012). Obesity is a risk factor for musculoskeletal pain in adolescents:
Findings from a population-based cohort. Pain.

[23] Stone AA, Broderick JE. (2012). Obesity and pain are associated in the united
states. Obesity (Silver Spring).

[24] Shiri R, Solovieva S, Husgafvel-Pursiainen K, Telama R, Yang X, Viikari J (2013). The role of obesity and physical activity in nonspecific and
radiating low back pain: The young finns study. Semin Arthritis Rheum.

[25] Da Rosa BN, Secrieru J, Candotti CT. (2021). Analysis of postural asymmetry on sagittal plane between right
and left side views using photogrammetry. J Bodyw Mov Ther.

[26] Bosso LR, Golias ARC. (2012). A postura de atletas de ginástica rítmica:
análise através da fotometria. Rev Bras Med Esporte.

[27] Baroni BM, Bruscatto CA, Rech RR, Trentin L, Brum LR. (2010). Prevalência de alterações posturais em
praticantes de musculação. Fisioter Mov.

[28] Hori Y, Hoshino M, Inage K, Miyagi M, Takahashi S, Ohyama S (2019). ISSLS Prize in clinical science 2019: Clinical importance of
trunk muscle mass for low back pain, spinal balance, and quality of life-a
multicenter cross-sectional study. Eur Spine J.

[29] Sadler SG, Spink MJ, Ho A, De Jonge XJ, Chuter VH. (2017). Restriction in lateral bending range of motion, lumbar lordosis,
and hamstring flexibility predicts the development of low back pain: A
systematic review of prospective cohort studies. BMC Musculoskelet Disord.

[30] Mundal I, Gråwe RW, Bjørngaard JH, Linaker OM, Fors EA. (2014). Prevalence and long-term predictors of persistent chronic
widespread pain in the general population in an 11-year prospective study:
The hunt study. BMC Musculoskelet Disord.

